# Structural Investigation of Poly(ethylene furanoate) Polymorphs

**DOI:** 10.3390/polym10030296

**Published:** 2018-03-09

**Authors:** Lucia Maini, Matteo Gigli, Massimo Gazzano, Nadia Lotti, Dimitrios N. Bikiaris, George Z. Papageorgiou

**Affiliations:** 1 Department of Chemistry “G. Ciamician”, Via Selmi 2, University of Bologna, 40126 Bologna, Italy; l.maini@unibo.it; 2 Department of Chemical Science and Technologies, University of Roma Tor Vergata, Via della Ricerca Scientifica 1, 00133 Roma, Italy; matteo.gigli@uniroma2.it; 3 Civil, Chemical, Environmental and Materials Engineering Department, University of Bologna, Via Terracini 28, 40131 Bologna, Italy; nadia.lotti@unibo.it; 4 Organic Synthesis and Photoreactivity Institute, ISOF-CNR, Via Gobetti 101, 40129 Bologna, Italy; 5 Laboratory of Polymer Chemistry and Technology, Department of Chemistry, Aristotle University of Thessaloniki, Thessaloniki 54124, Greece; dbic@chem.auth.gr; 6 Chemistry Department, University of Ioannina, P.O. Box 1186, Ioannina 45110, Greece; gzpap@cc.uoi.gr

**Keywords:** poly(ethylene furanoate), PEF, 2,5-furan dicarboxylate, crystal structure, polymorphism

## Abstract

α and β crystalline phases of poly(ethylene furanoate) (PEF) were determined using X-ray powder diffraction by structure resolution in direct space and Rietveld refinement. Moreover, the α’ structure of a PEF sample was refined from data previously reported for PEF fiber. Triclinic α-PEF *a* = 5.729 Å, *b* = 7.89 Å, *c* = 9.62 Å, α = 98.1°, β = 65.1°, γ = 101.3°; monoclinic α’-PEF *a* = 5.912 Å, *b* = 6.91 Å, *c* = 19.73 Å, α = 90°, β = 90°, γ = 104.41°; and monoclinic β-PEF *a* = 5.953 Å, *b* = 6.60 Å, *c* = 10.52 Å, α = 90°, β = 107.0°, γ = 90° were determined as the best fitting of X-ray diffraction (XRD) powder patterns. Final atomic coordinates are reported for all polymorphs. In all cases PEF chains adopted an almost planar configuration.

## 1. Introduction

In recent years, in view of a greener and more sustainable economy, many efforts from both academic and industrial sectors have been devoted to the development of bio-based alternatives to fossil-based plastics. Bioplastic production is expected to increase from the actual 4.2 million tons to over 6.1 million tons in 2021, thus highlighting a very fast growing rate [1].

Among different renewable starting materials that have been used for the preparation of bioplastics, furan-based monomers have attracted considerable attention. In particular, 2,5-furandicarboxylic acid (FDCA), whose initial diffusion was hampered by the difficulty to produce large amounts with high purity, has been lately the object of much research [2,3]. Currently, FDCA can be readily obtained from the oxidation of hydroxymethylfurfural, in turn derived from the dehydration of (poly)saccharides or by exploiting new synthetic routes, such as through 2-furoic acid and CO_2_ [4,5,6]. From this framework, in October 2016, the Dutch company Avantium announced the establishment of a new joint venture with BASF corporation, named Synvina, for the large-scale production and marketing of FDCA [7].

The reason behind the success of FDCA mostly lies in its use for the synthesis of poly(ethylene-2,5-furanoate) (PEF), considered the most credible bio-based alternative to poly(ethylene terephthalate) (PET), thanks to its very interesting physical/mechanical and barrier properties. PEF can be processed with outstanding results into films, fibers, and, above all, bottles for beverage packaging. From a comparison between the barrier properties of PEF and PET, the following outcomes emerged: PEF exhibited 11× reduction in oxygen permeability [8], 19× reduction in carbon dioxide permeability [9], and 5× lower water diffusion coefficient [10] compared to PET. In addition, PEF displays more attractive thermal and mechanical properties than PET: higher *T*_g_ (85 °C vs. 76 °C), lower *T*_m_ (211 °C vs. 247 °C) [11], and 1.6× higher Young’s modulus [2]. Lastly, the production of PEF would decrease non-renewable energy use by about 40–50% and greenhouse gas emissions by ca. 45–55% with respect to PET [12].

Several papers in the literature have been devoted to PEF characterization [10,13,14]. The relationships between the thermal and structural properties of PEF have been investigated to explain the complex behavior exhibited during isothermal crystallization. Two phases, called α’ and α, were identified by Stoclet et al. [15]. The influence of different experimental conditions on the crystallization, stability, and transformation of PEF polymorphs were reported by Tsanatkis et al.; they also described a new β-phase obtained after solvent crystallization [16,17].

Nevertheless, the crystal structures of PEF polymorphs have yet to be defined, and further investigation is therefore necessary. Indeed, the triclinic cell proposed in an early study [18] with dimensions of *a* = 5.75 Å, *b* = 5.35 Å, *c* = 20.1 Å, α = 113.5°, β = 90°, and γ = 112° is not compatible with the data reported by all other authors whatever PEF phase is considered, as it would cause a wrong positioning of the main peaks in the XRD pattern. Although in a recent work by Mao et al. [19] the high-resolution structure of a PEF fiber is documented, a comparison between the powder and fiber data is needed. During the review process of this paper, the structural evolution of PEF upon uniaxial stretching was deeply investigated. Evidence of a mesomorphic phase at low stretching temperatures and of a crystalline phase, different from the thermally induced ones, was reported [20]. Therefore, the main aim of the present work is to shed light on the crystal structures of α-, α’-, and β-PEF obtained during usual PEF preparations, with no need for fibers or stretching conditions, from powder pattern X-ray diffraction. The study of structure-property relationships is indeed of crucial importance for the optimization of the end-use behavior of a polymeric material.

## 2. Materials and Methods

### 2.1. Materials and Sample Preparation

2,5-furan dicarboxylic acid (purum 97%), ethylene glycol, and tetrabutyl titanate catalyst of analytical grade were purchased from Sigma-Aldrich Co. (St. Louis, MO, USA) All other materials and solvents used were of analytical grade. Poly(ethylene furandicarboxylate) polyester was synthesized as previously reported by the two-stage melt polycondensation method (esterification and polycondensation) in a glass batch reactor [21,22].

**Scheme 1:**
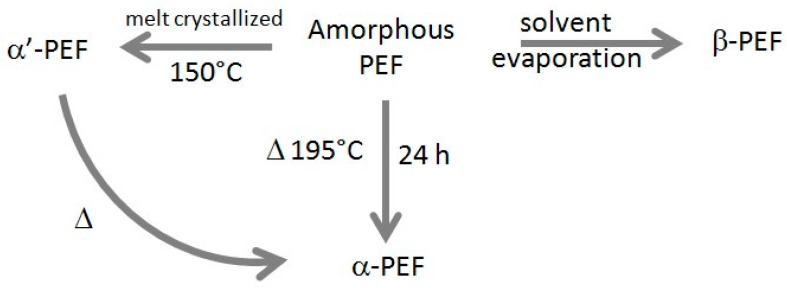
Preparation of different crystal forms of poly(ethylene-2,5-furanoate) PEF.

The PEF sample displays an intrinsic viscosity value 0.45 dL/g. Its weight- and number-average molecular weight (*M*_w_, *M*_n_), measured by gel permeation chromatography (GPC) apparatus equipped with differential refractometer as detector (Waters Inc., Milford, MA, USA), are respectively *M*_w_ = 24,640 g/mol and *M*_n_ = 11,200 g/mol (Polydispersity Index, PDI = 2.2). To attain a high degree of crystallinity and to favor the selective formation of pure crystalline phases, α-PEF was obtained by annealing an “as-synthesized” sample at 195 °C for 24 h, α’-PEF was obtained by isothermal crystallization at 150 °C of a melt-quenched sample, and β-PEF was obtained by slow solvent evaporation at room temperature after dissolution in a trifluoroacetic acid/chlorofom 1/5 *v*/*v* mixture and precipitation in cold methanol. An overall sample preparation procedure is reported in Scheme 1.

### 2.2. Diffraction and Infrared Measurements

X-ray diffraction (XRD) patterns were collected in Bragg-Brentano geometry with a flat sample holder, over the 2θ range 3°–80° (40 kW–40 mA; Cu-Kα radiation λ = 1.5418 Å, step size 0.05°, 1500 s/step) on a X’Pert PRO automated diffractometer (Panalytical, Almelo, The Netherlands) equipped with a fast X’Celerator detector. Crystallinity was determined as X_c_ = A_c_/A_tot_, where A_c_ represents the integrated crystalline scattering and A_tot_ the integrated total scattering, both crystalline and amorphous; air and incoherent scattering were taken in due consideration. Attenuated Total Reflectance Fourier Transform Infrared (ATR-FTIR) spectra were recorded on an Alpha FT IR spectrometer (Bruker Optik GmbH, Ettlingen, Germany) with a platinum ATR single reflection diamond module. The background spectrum of air was collected before the acquisition of each sample spectrum. Spectra were recorded with a resolution of 2 cm^−1^, and 64 scans were averaged for each spectrum (scan range 4000–450 cm^−1^).

### 2.3. Structure Determinations

Topas 5 software package (Coelho Software, Brisbane, Australia) was used for indexing and structure determination in direct space and Rietveld refinement. A Pawely refinement was performed with the best cells, and the best one for each phase was chosen for structure solution. It was run in direct space by a simulated annealing algorithm. During the solution, the two torsional angles of the monomer were allowed to move freely. The background was described by the Chebyshev function with four parameters, while the amorphous content was modeled with two peaks centered at 2θ = 22.1° and 45.8° based on a fully amorphous PEF profile, and suitably scaled on each pattern of α-, α’-, β-PEF, respectively. The peak profile was described by a combination of Gaussian and Lorentzian functions. Microstructural parameters are reported in Table S1 (Supplementary Materials).

## 3. Results and Discussion

### 3.1. Sample Characterization

**Figure 1:**
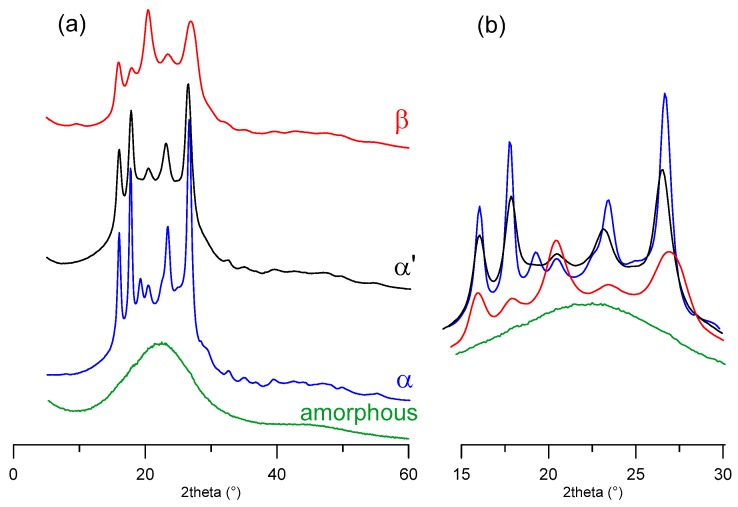
X-ray diffraction (XRD) patterns of PEF: (**a**) from top to bottom: β-, α’-, α-phase and amorphous sample; (**b**) superposition of the patterns in the range 15°–30°.

To the best of our knowledge, PEF displays three different XRD patterns [16,17]. Various experimental conditions were screened to obtain highly crystalline samples showing pure crystal phases. The diffraction patterns of the samples, used for structure resolution and refinements, are reported in Figure 1 together with the profile of an amorphous ‘as-synthesized’ sample. The degrees of crystallinity, as measured by XRD, are 47, 45, and 27% for α, α’, and β samples, respectively. α and α’ profiles are very similar, although α-PEF shows sharper reflections and an extra peak at 19.3° (2θ). On the other hand, β-PEF exhibits five main reflections roughly positioned at the same angular values as α’-PEF. However, they are broader and display completely different relative intensities. Lastly, an additional low-intensity reflection is detectable at 9.5°.

The Temperature Modulated Differential Scanning Calorimetry (TMDSC) scans reported in Figure S1 show the presence of narrow melting peaks for the solution crystallized sample (β crystals) and for the sample crystallized at 195 °C (α crystals). In particular, in the non-reversing signal curves, only a large non-reversing melting appeared, indicating the high perfection and thermal stability of the crystals, which do not suffer any recrystallization/reorganization upon heating. This result is consistent with the highly stable unique crystalline phases. For the sample crystallized at 170 °C a small recrystallization peak appeared, but only after non-reversing melting and at a very high temperature, just before the end of melting. On the other hand, PEF crystallized at 150 °C, showed a broad melting. A recrystallization exothermic peak was also observed above 170 °C, in the non-reversing signal curve, as well as in the case of the melt-quenched sample. This behavior indicates that crystal perfection occurred upon heating, due to the poor nature of the original crystals. This behavior is consistent with the transition from the less perfect α’ to the α crystal phase at 170 °C, as already reported in the literature [15,16,17], Scheme 1. The ATR-FTIR spectra analysis, reported in Figure S2, confirm the presence of the right functional groups of the polymer (comparisons in Figure S3), but do not provide any specific insight about the solid phase, since no particular differences were detected in the bands position. A careful analysis of XRD data can give further confidence about the phase purity. As can be seen in Figure 1b, the peak at 19.3° (2θ) can be taken as a α-phase marker because at this angular value both of the other two samples display no peaks, thus indicating a complete absence of the α-phase. Similarly, a verification of the base line intensity around 9.5° in the α’-, α-samples excludes the presence of the β-phase because of the detection of a flat background.

### 3.2. Crystal Solutions and Refinements

Structural analysis was carried out on powder patterns and each dataset was treated as completely new. Patterns were indexed using 16 peaks and the cells were chosen based on cell volume and calculated density. Cells with a density higher than 1.60 (g/cm^3^) or with a volume corresponding to a non-integer number of molecules were discarded. 

**Figure 2:**
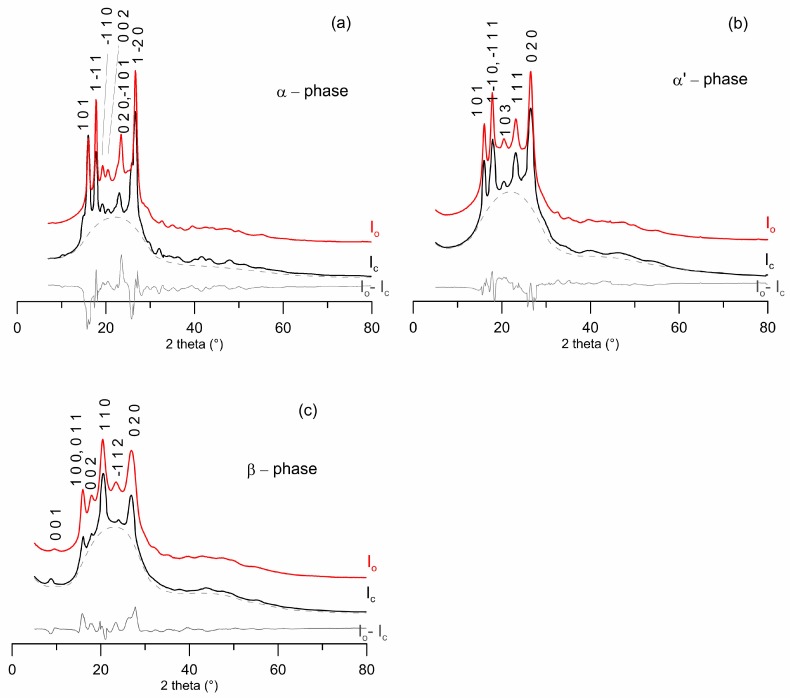
Comparison between observed (red), calculated (black), and Io-Ic difference (grey) powder-diffraction patterns: (**a**) α-PEF; (**b**) α’-PEF; (**c**) β-PEF. Dashed line (grey) represents the sum of background and amorphous phase contributions. The Miller indices of the reflections that mainly contribute to the peak intensities are reported.

The values found, although unusually high for organic polymers, were not surprising since comparably high densities for PEF have been reported in the literature [11,19]. A pretty good agreement between the observed and calculated diffraction profiles was obtained for all of the polymorphs (Figure 2). Cell parameters and discrepancy factors are reported in Table 1. The R_p_ factors achieved, lower than 13%, are satisfactory for polymer structures determined by powder diffraction. The fractional atomic coordinates of the asymmetric unit for each of the phases are reported in Tables S2–S4, and the atomic distances and angles are reported in Tables S5–S7.

### 3.3. α-PEF

**Figure 3:**
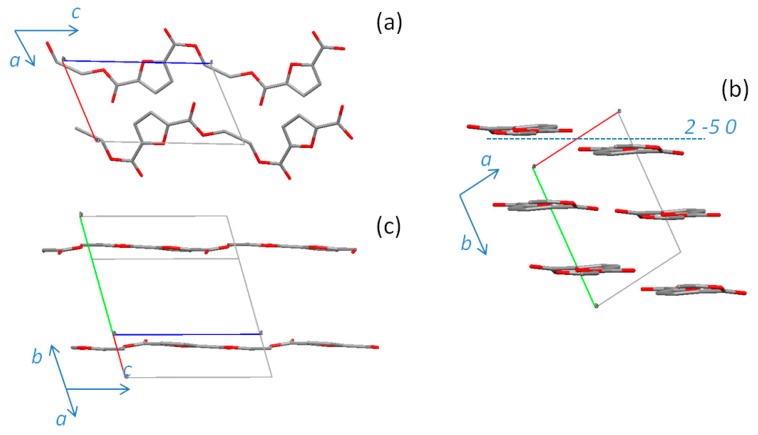
Views of the chain arrangements in the unit cell for the α-PEF structure: (**a**) along the *b*-axis; (**b**) projection down the *c*-axis; (**c**) a view to highlight chain section. Oxygen atoms are in red; dotted line represents the intersection of the *2 -5 0* plane with the *a*, *b* plane.

The structure of α-PEF is displayed in Figure 3. The unit cell contains two monomers. The polymer chain expands in opposite directions with respect to the furanic ring due to the orientation of the two ester groups. The chains are aligned in the *c*-axis direction and lie roughly parallel to the *2 -5 0* plane (see Figure 3b,c).

### 3.4. α’-PEF

**Figure 4:**
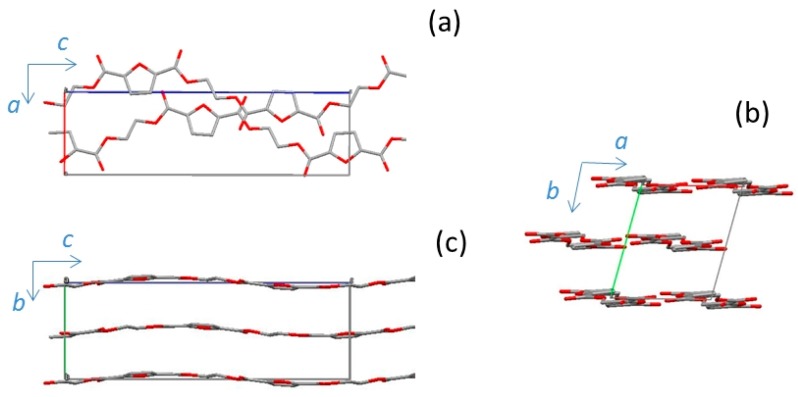
Views of the chain arrangement for the α’-PEF structure: (**a**) projection down the *b*-axis; (**b**) along the *c*-axis; (**c**) projection down the *a*-axis. Oxygen atoms are in red.

The less stable α’-phase shows a different structure. During the course of this study the work of Mao et al. appeared in the literature, reporting the PEF structure of a fiber sample investigated with synchrotron light [19]. We found that one of the proposed structures showed a calculated powder pattern very similar to that of α’-PEF except for the amorphous “bump”, see Figure S4. For this reason, we refined the α’-PEF phase starting from the data of the 3/12 structure, as proposed by Mao et al. With respect to the cell determined from a fiber sample [19], we found longer *a* and *b* axes and a shorter *c* axis. The pseudo monoclinic unit cell displays a double volume as compared to α-PEF and hosts four molecular units organized in two chains. Each of these has two monomers in a “trans” planar conformation with a 2_1_ axis between adjacent monomers, as shown in Figure 4. The two chains in the unit cell are parallel to the *a*, *c* plane, staggered by 3/12 of the *c*-axis in the chain direction, and slightly bent. 

### 3.5. β-PEF

**Figure 5:**
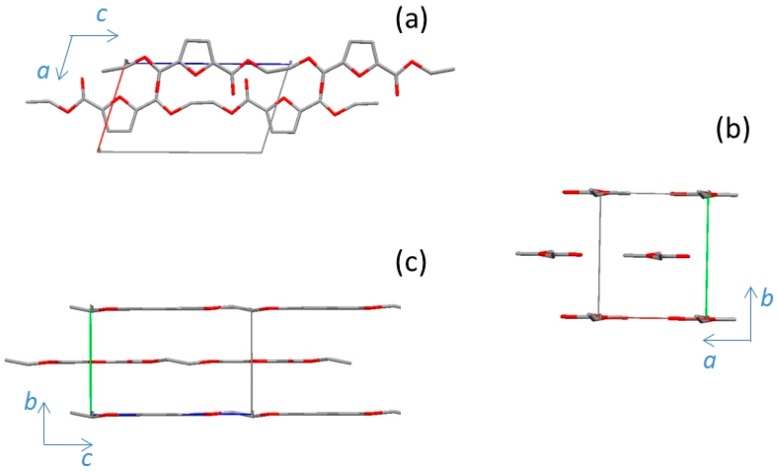
Views of the chain packing for the β-PEF structure: (**a**) along the *b*-axis; (**b**) projection down the *c*-axis; (**c**) projection down the *a*-axis. Oxygen atoms are in red.

The crystal structure of the β-phase, generated by solvent-induced crystallization, is displayed in Figure 5. Two planar molecules are contained in the monoclinic cell with a volume slightly bigger than that in the α-phase. Two flat chains lie parallel to the *a*, *c* plane, shifted 0.4 units in the *c*-axis direction. 

Taking in the due consideration the thermal expansion of the unit cell, β-PEF may also be a good candidate for comparison with the structure of the strain-induced phase followed by in situ heat treatment, as reported by Stoclet et al. [20].

## 4. Conclusions

Suitable experimental conditions to obtain PEF samples with a unique crystal phase and very good crystallinity have been found. By structure determination in direct space (α-, β-phases) or by structure refinement (α’-phase), the solutions that gave the best fit with the experimental X-ray powder patterns have been determined. The unusual high density values of PEF have been confirmed and justified. The structure previously reported for a PEF fiber corresponds to the α’-phase and shows a double cell volume as compared to the other forms. None of the PEF phases is isomorphous to PET crystal lattice. PEF structures show unit cell dimensions different from PET [23]; nevertheless, the chain packing in α-PEF is similar to that of the polymer derived from terephthalic acid (see Figure S5). The presence of several polymorphs for PEF, as compared to the unique phase so far known for PET, could be associated with different factors, such as the intrinsic lower symmetry of furane with respect to benzene, which causes much more conformations, and to the lower chain mobility and higher rigidity of furanoate with respect to terephthalate [11].

## Figures and Tables

**Table 1:** Structural data; estimated standard deviations (e.s.d.) in parentheses.

Cell Parameters	α-PEF	α’-PEF	β-PEF
a (Å)	5.729(7)	5.912(3)	5.953(3)
b (Å)	7.89(3)	6.913(4)	6.600(3)
c (Å)	9.62(6)	19.73(2)	10.52(1)
α (°)	98.1(3)	90.0	90.0
β (°)	65.1(4)	90.0	107.0(1)
γ (°)	101.3(4)	104.41(3)	90.0
S.G.	P-1	P1	P1
Vol (Å^3^)	385.85	780.84	394.30
ρ_calc_ (g/cm^3^)	1.567	1.549	1.482
R_p_ (%)	12.5	11.4	13.2
R_wp_ (%)	13.2	12.1	13.5

## Supplementary Materials

The following are available online at http://www.mdpi.com/2073-4360/10/3/296/s1, Figure S1: TMDSC scans, Figure S2: ATR-FTIR scans, Figure S3: comparisons of IR spectra, Figure S4: calculated pattern of 3/12 structure reported by Mao, Figure S5: overlap of the crystal structures of PET and α-PEF, Table S1: microstructural parameters, Table S2: crystal data of α-PEF, Table S3: crystal data of α’-PEF, Table S4: crystal data of β-PEF, Table S5: bond distances and angles of α-PEF, Table S6: bond distances and angles of α’-PEF, Table S7: bond distances and angles of β-PEF.
