# Supplementary material for: Structural Investigation of Poly(ethylene furanoate) Polymorphs
Source: Polymers (Basel). 2018 Mar 9;10(3):296. doi: 10.3390/polym10030296 (PMC6415112; doi:10.3390/polym10030296)
Supplement: Supplementary file 1 [file polymers-10-00296-s001.pdf]

Supplemetary Materials

Structural Investigation of Poly(ethylene furanoate) Polymorphs

Contents

Figure S1	TMDSC scans	Pag.	2
Figure S2	ATR-FTIR scans		3
Figure S3	IR comparisons		4
Figure S4	Calculated XRD from Mao structure		5
Figure S5	PET / PEF structure comparisons		5
Table S1	Microstructural parameters		6
Table S2	Crystal data of α-PEF		6
Table S3	Crystal data of α'-PEF		7
Table S4	Crystal data of β-PEF		9
Table S5	Bond distances and angles of α-PEF		10
Table S6	Bond distances and angles α'-PEF		11
Table S7	Bond distances and angles β-PEF		13

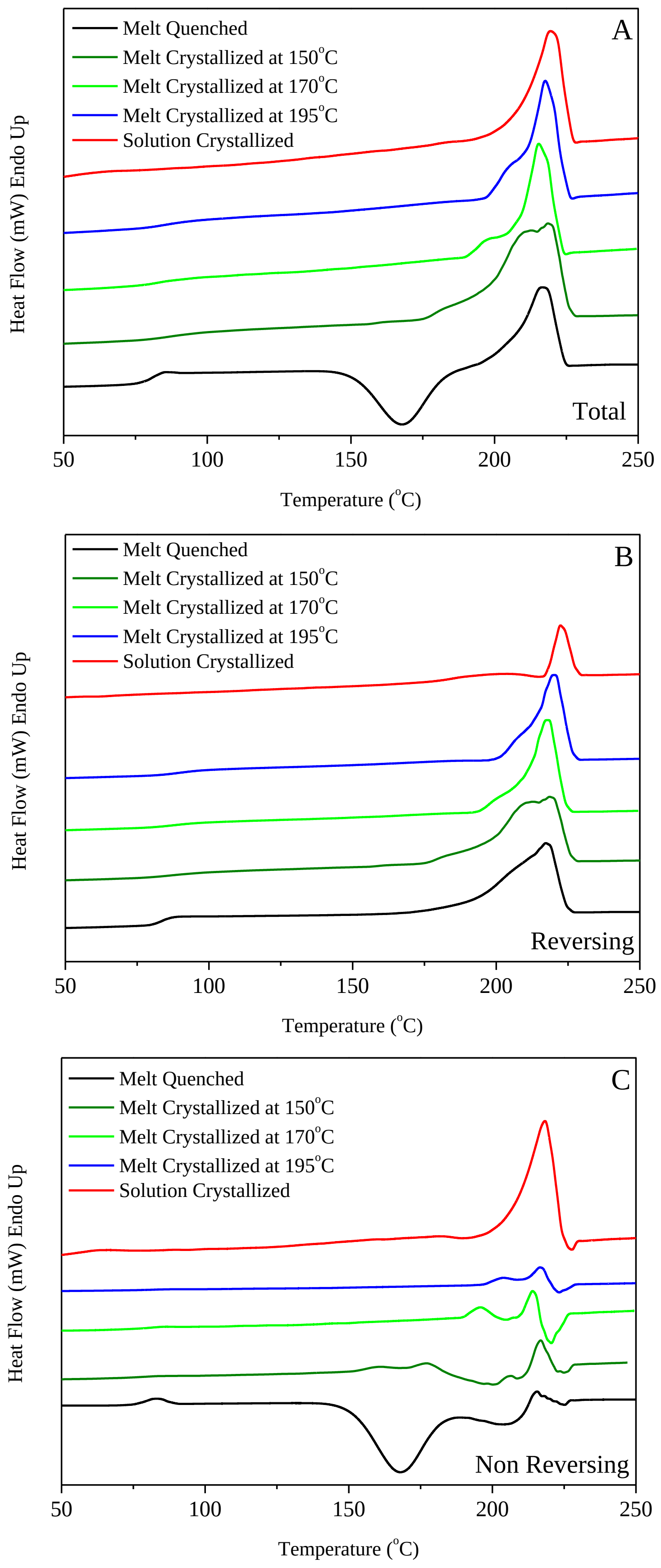

Figure S1: TMDSC data for solution crystallized, melt crystallized at 195°C, 170°C and 150°C and melt quenched PEF: A) Total, B) Reversing and C) Non Reversing signal.

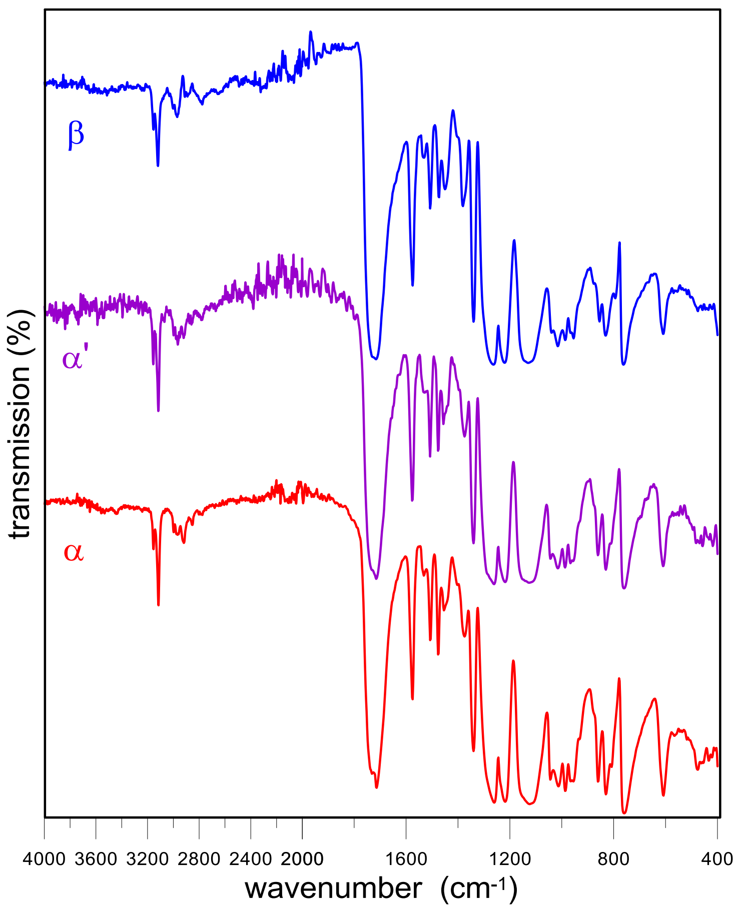

Figure S2. ATR-FTIR spectra of α, α' and β crystalline phases of PEF.

Comment: two stretching-vibration bands of aromatic C-H at 3155 and 3116 cm^{-1} and several partially overlapped bands between 3000 and 2850 cm^{-1} due to aliphatic C-H can be observed. A large intense signal characteristic of stretching vibration centered at 1720 cm^{-1} for β-PEF, double tipped at 1733 and 1715 cm^{-1} in α and α'-PEF, is also visible. These frequencies are typical for C=O stretching of α,β conjugated esters and are close to the values reported for diethyl 2,5-furandicarbossilate and PET. The band at 1577 cm^{-1} is due to aromatic C=C stretching. In the fingerprint region, the similarity of PEF spectrum with the furandicarboxylate one is poorer and its better with PET spectrum (see Figure S3). Indeed, the presence of semicrystalline polymeric material increases the band widths, as found in PET.

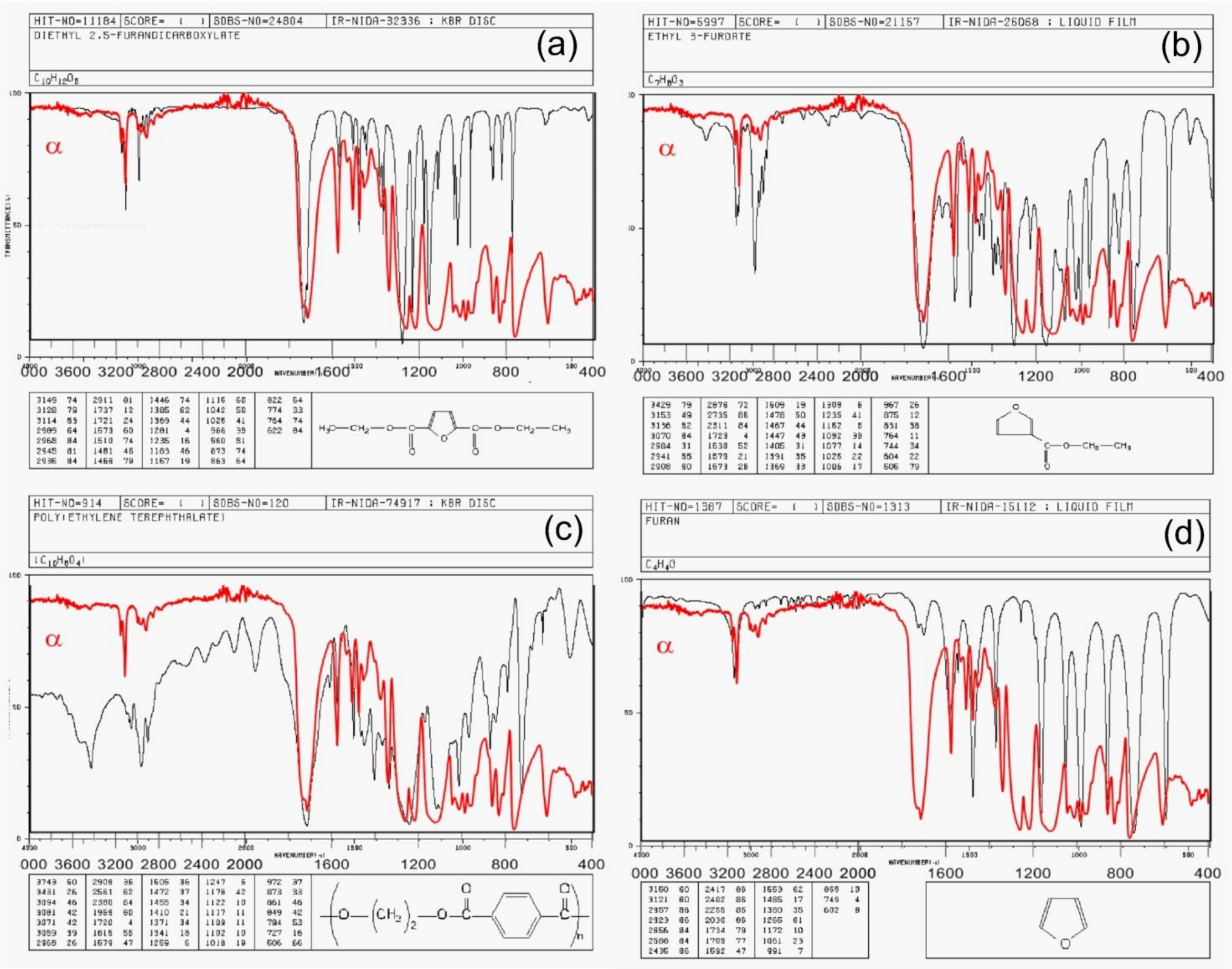

Figure S3: overlap of α-PEF spectrum, ATR-FTIR mode, with diethyl 2,5-furandicarboxylate, KBr disc (a), ethyl 3-furoate, liquid film (b), PET, KBr disc (c), furan, liquid film (d).

Reference: National Institute of Advanced Industrial Science and Technology, SDBSWeb (Accessed on 31 October 2017) : http://sdbs.db.aist.go.jp

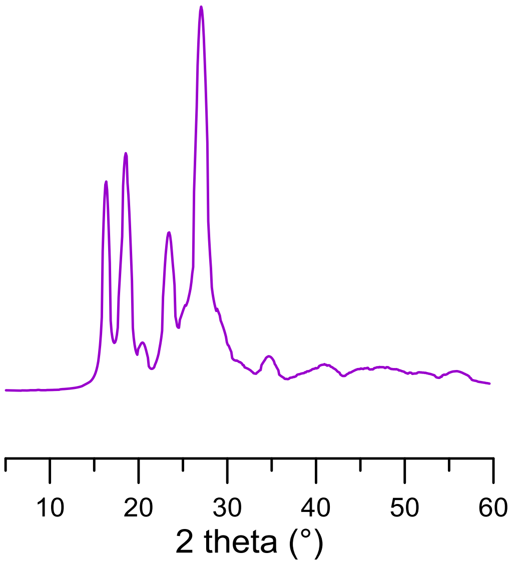

Figure S4: XRD pattern calculated from the data of 3/12 structure reported by Mao, Y.; Kriegel, R.M. and Bucknall, D.G. *Polymer* 2016, 102, 308-314.
https://doi.org/10.1016/j.polymer.2016.08.052

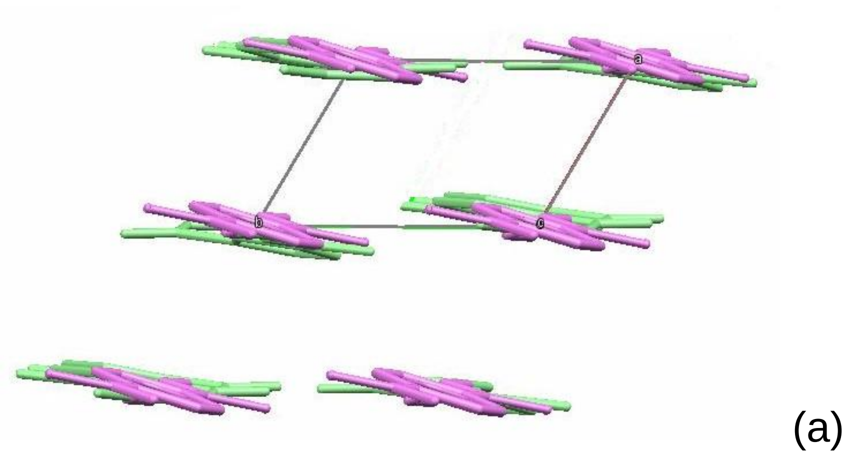

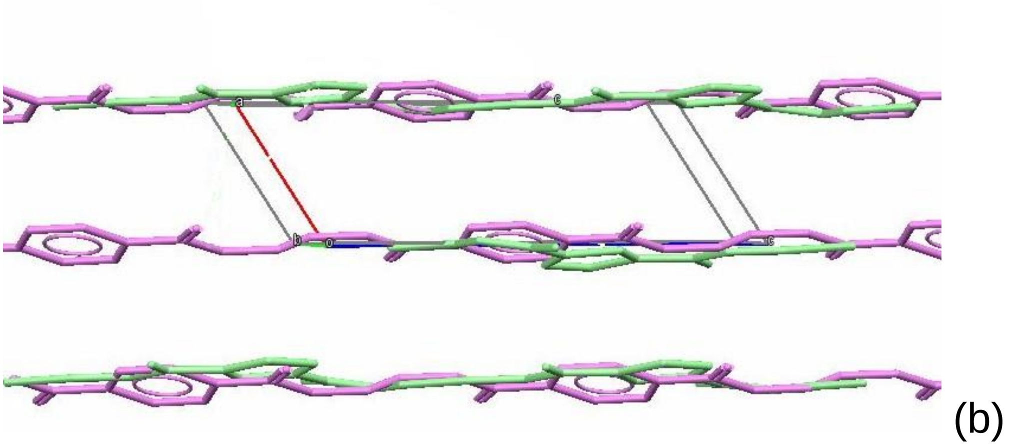

Figure S5: Overlap of the crystal structure of PET (cyan) and α-PEF (light green); (a) view along the chains, (b) longitudinal view. The PET unit cell is shown.

Table S1: microstructural parameters

PEF phase	Preferenzial Orientation *	Isotropic Crystal size (nm)
α	Toraya plate *0 0 2* P_1=0.3 P_2=0.0	15 ±2
α'	n.d.	12±2
β	Toraya plate *1 0 0* P_1=1.1 P_2=2.2	9±2

* Toraya, H. and Marumo, F. Preferred orientation correction in powder pattern-fitting. *Mineral. J.* 1981, *10*, 211-221 https://doi.org/10.2465/minerj.10.211

Preferred orientation was corrected by the function: $p(\phi)=P_1 + (1-P_1) * \exp(-P_2*\phi^2)$ where ϕ is the acute angle between preferred orientation direction and scattering vector, P_1 and P_2 are fitting parameters.
Crystal size was estimated as an isotropic parameter in order to give comparison between the samples. It is obtained by the mean value of the FWHM (full width at half maximum) of the three most intense reflections for each phase by Scherrer equation [1].
$C.S._{hkl} = K\lambda / (b_{1/2}*\cos\theta)$ where λ is the wavelength, $b_{1/2}$ the $FWHM_{hkl}$, θ the diffraction angle and K a constant depending on crystal habit (chosen as 1.0). The silicon standard peak 111 was used to evaluate the instrumental broadening.
[1] Klug, H.P., Alexander, L.E. X-ray diffraction procedures for polycrystalline and amorphous materials. New York: Wiley Interscience; 1974.

Table S2: Crystal data of α-PEF

TITL	AlfaApr A-C	*a*	*b*	*c*	α	β	γ
CELL		5.729	7.893	9.61726	98.063	65.0742	101.345
		x	y	z	O.F.	U	
O1		0.01189	0.27039	0.45026	1	0.006333	
C1		0.00615	0.28459	0.31154	1	0.006333	
C2		-0.22708	0.18317	0.53826	1	0.006333	
C3		0.23502	0.37116	0.18815	1	0.006333	
C4		-0.22712	0.20831	0.31263	1	0.006333	
C5		-0.29109	0.14913	0.6972	1	0.006333	
C6		-0.3772	0.14355	0.45696	1	0.006333	
O2		0.21767	0.33969	0.05229	1	0.006333	
O3		0.41324	0.4586	0.21088	1	0.006333	
O4		-0.10413	0.21718	0.74591	1	0.006333	
O5		-0.49739	0.06792	0.77458	1	0.006333	
C7		-0.01601	0.23609	0.04167	1	0.006333	
C8		-0.15901	0.18329	0.90267	1	0.006333	
S.G. P-1							

Table S3: Crystal data of α'-PEF

TITL	3/12 refined	*a*	*b*	*c*	α	β	γ
CELL		5.9123	6.9126	19.7259	90	90	104.406
		x	y	z	O.F.	U	
C1	1	-0.0679	0.0437	0.0333	1	0.05	
O2	2	-0.0573	0.0002	0.1022	1	0.05	
C3	1	-0.2522	-0.0029	0.1404	1	0.05	
C4	1	-0.1971	-0.0297	0.2104	1	0.05	
C5	1	0.0176	-0.0544	0.2333	1	0.05	
C6	1	0.0021	-0.0672	0.3022	1	0.05	
C7	1	-0.2218	-0.0506	0.3199	1	0.05	
O8	2	-0.3447	-0.0284	0.2635	1	0.05	
H9	3	0.1745	-0.0615	0.204	1	0.05	
H10	3	0.1448	-0.0867	0.3352	1	0.05	
C11	1	-0.3068	-0.0487	0.3885	1	0.05	
O12	2	-0.5046	-0.0478	0.4081	1	0.05	
O13	2	-0.1245	-0.0451	0.4313	1	0.05	
C14	1	-0.1588	-0.0268	0.5008	1	0.05	
O15	2	-0.4379	0.0162	0.1165	1	0.05	
H16	3	-0.198	0.1205	0.5127	1	0.05	
H17	3	-0.0966	0.1966	0.0257	1	0.05	
H18	3	-0.3087	-0.1451	0.5188	1	0.05	
H19	3	-0.2168	-0.0619	0.0093	1	0.05	
C39	1	0.0679	-0.0437	0.5333	1	0.05	
O40	2	0.0573	-0.0002	0.6022	1	0.05	
C41	1	0.2522	0.0029	0.6404	1	0.05	
C42	1	0.1971	0.0297	0.7104	1	0.05	
C43	1	-0.0176	0.0544	0.7333	1	0.05	
C44	1	-0.0021	0.0672	0.8023	1	0.05	
C45	1	0.2218	0.0506	0.8199	1	0.05	
O46	2	0.3447	0.0284	0.7635	1	0.05	
H47	3	-0.1745	0.0615	0.704	1	0.05	
H48	3	-0.1448	0.0867	0.8352	1	0.05	
C49	1	0.3068	0.0487	0.8885	1	0.05	
O50	2	0.5046	0.0478	0.9081	1	0.05	
O51	2	0.1245	0.0451	0.9312	1	0.05	
C52	1	0.1588	0.0268	0.0008	1	0.05	
O53	2	0.4379	-0.0162	0.6165	1	0.05	
H54	3	0.198	-0.1205	0.0127	1	0.05	
H55	3	0.0966	-0.1966	0.5257	1	0.05	
H56	3	0.3087	0.1451	0.0188	1	0.05	
H57	3	0.2168	0.0619	0.5094	1	0.05	
C20	1	0.3412	0.4731	0.2503	1	0.05	
O21	2	0.3755	0.4549	0.3199	1	0.05	
C22	1	0.1931	0.4512	0.3626	1	0.05	

C23	1	0.2782	0.4494	0.4312	1	0.05
C24	1	0.5021	0.4329	0.4489	1	0.05
C25	1	0.5177	0.4457	0.5178	1	0.05
C26	1	0.3029	0.4704	0.5407	1	0.05
O27	2	0.1553	0.4717	0.4876	1	0.05
H28	3	0.6449	0.4133	0.4159	1	0.05
H29	3	0.6746	0.4386	0.5471	1	0.05
C30	1	0.2478	0.4972	0.6107	1	0.05
O31	2	0.0622	0.5164	0.6346	1	0.05
O32	2	0.4428	0.5003	0.6489	1	0.05
C33	1	0.4322	0.5438	0.7178	1	0.05
O34	2	-0.0046	0.4522	0.3431	1	0.05
H35	3	0.4035	0.6967	0.7254	1	0.05
H36	3	0.302	0.6204	0.2384	1	0.05
H37	3	0.2832	0.4382	0.7418	1	0.05
H38	3	0.1913	0.3548	0.2324	1	0.05
C58	1	0.6588	0.5269	0.7503	1	0.05
O59	2	0.6245	0.5451	0.8199	1	0.05
C60	1	0.8069	0.5488	0.8626	1	0.05
C61	1	0.7218	0.5505	0.9312	1	0.05
C62	1	0.4979	0.5671	0.9489	1	0.05
C63	1	0.4823	0.5543	0.0178	1	0.05
C64	1	0.6971	0.5296	0.0407	1	0.05
O65	2	0.8447	0.5283	0.9876	1	0.05
H66	3	0.3552	0.5867	0.9159	1	0.05
H67	3	0.3254	0.5614	0.0471	1	0.05
C68	1	0.7522	0.5027	0.1107	1	0.05
O69	2	0.9378	0.4836	0.1347	1	0.05
O70	2	0.5573	0.4997	0.1489	1	0.05
C71	1	0.5678	0.4562	0.2178	1	0.05
O72	2	1.0046	0.5478	0.8431	1	0.05
H73	3	0.5965	0.3033	0.2254	1	0.05
H74	3	0.698	0.3796	0.7384	1	0.05
H75	3	0.7168	0.5618	0.2418	1	0.05
H76	3	0.8087	0.6452	0.7324	1	0.05

S.G.1

Table S4: Crystal data of β-PEF

TITL	BETA	*a*	*b*	*c*	α	β	γ
CELL		5.95	6.6	10.5	90	107	90
		x	y	z	O.F.	U	
C(2)		0.0904	1.02	0.8813	1	0.05	
O(1		-0.0389	1	0.7421	1	0.05	
C(3		0.1126	1	0.6705	1	0.05	
C(4		-0.0142	1	0.5344	1	0.05	
C(5		-0.2535	1	0.4639	1	0.05	
C(6		-0.2616	1	0.3292	1	0.05	
O(2		0.3371	1	0.7161	1	0.05	
C(7		-0.0268	1	0.326	1	0.05	
O(3		0.1266	1	0.4512	1	0.05	
C(8		0.0896	1	0.2255	1	0.05	
O(5		-0.0702	1	0.1083	1	0.05	
C(9		0.0441	0.98	0.0092	1	0.05	
O(4		0.3129	1	0.2455	1	0.05	
C(2)		0.4447	0.52	1.4771	1	0.05	
O(1)		0.566	0.5	1.3754	1	0.05	
C(3)		0.4082	0.5	1.2575	1	0.05	
C(4)		0.5271	0.5	1.1582	1	0.05	
C(5)		0.7641	0.5	1.159	1	0.05	
C(6)		0.7633	0.5	1.0255	1	0.05	
O(2)		0.1845	0.5	1.2361	1	0.05	
C(7)		0.5259	0.5	0.9515	1	0.05	
O(3)		0.3794	0.5	1.0319	1	0.05	
C(8)		0.4016	0.5	0.8151	1	0.05	
O(5)		0.5551	0.5	0.7447	1	0.05	
C(9)		0.433	0.48	0.6102	1	0.05	
O(4)		0.1773	0.5	0.768	1	0.05	
S.G.1							

Table S5: Main bond distances and angles of α-PEF structure

atom1	atom2	distance
O1	C1	1.3689
C1	C3	1.4689
C2	C5	1.4653
C2	C6	1.3569
C3	O2	1.337
C3	O3	1.198
C4	C6	1.3955
C5	O4	1.3346
C5	O5	1.2066
O4	C8	1.4573

atom1	atom2	atom3	angle
O1	C1	C3	120.4102
C1	C3	O2	112.1174
C1	C3	O3	122.2783
C5	C2	C6	129.3015
C2	C5	O4	113.9683
C2	C5	O5	121.4888
C2	C6	C4	106.5793
O2	C3	O3	125.6041
O4	C5	O5	124.5383
C5	O4	C8	115.5176

Table S6: Main bond distances and angles of α'-PEF structure

atom1	atom2	distance
C1	O2	1.3968
C3	C4	1.4412
C4	O8	1.3648
C5	C6	1.3636
C7	O8	1.3582
C7	C11	1.4447
C11	O12	1.2332
C11	O13	1.3645
O13	C14	1.3962
C39	O40	1.3968
C41	C42	1.4412
C42	O46	1.3648
C43	C44	1.3656
C45	O46	1.3582
C45	C49	1.4447
C49	O50	1.2332
C49	O51	1.3632
C20	O21	1.3981
C20	C71	1.5157
O21	C22	1.3636
C22	C23	1.4448
C23	C24	1.4009
C23	O27	1.3584
C24	C25	1.3636
C25	C26	1.3978
C26	O27	1.3648
C26	C30	1.4412
C30	O31	1.2314
C30	O32	1.373
O32	C33	1.3968
C33	C58	1.5157
C58	O59	1.3981
O59	C60	1.3636
C60	C61	1.4448
C61	C62	1.4011
C61	O65	1.3582
C63	C64	1.3978
C64	C68	1.4414
C68	O69	1.232
C68	O70	1.3726
O70	C71	1.3967

atom1	atom2	atom3	angle
C3	C4	O8	124.6884
C4	O8	C7	105.4852
O8	C7	C11	124.6895
C7	C11	O12	128.7674
C7	C11	O13	107.7433
O12	C11	O13	123.4837
C11	O13	C14	118.5121
C41	C42	O46	124.6888
C42	O46	C45	105.4853
O46	C45	C49	124.6893
C45	C49	O50	128.7681
C45	C49	O51	107.6777
O50	C49	O51	123.5485
O21	C20	C71	104.9299
C20	O21	C22	118.4598
C20	C71	O70	107.9088
O21	C22	C23	107.6509
C22	C23	C24	124.9096
C22	C23	O27	124.6693
C24	C23	O27	110.3708
C23	C24	C25	107.0479
C23	O27	C26	105.4783
C24	C25	C26	106.3303
C25	C26	O27	110.7705
C25	C26	C30	124.5133
O27	C26	C30	124.6888
C26	C30	O31	128.0696
C26	C30	O32	108.1828
O31	C30	O32	123.7449
C30	O32	C33	117.0597
O32	C33	C58	107.8857
C33	C58	O59	104.9306
C58	O59	C60	118.4604
O59	C60	C61	107.6573
C60	C61	C62	124.8971
C60	C61	O65	124.6877
C62	C61	O65	110.3684
C63	C64	C68	124.5221
C64	C68	O69	128.1626
C64	C68	O70	108.1809
O69	C68	O70	123.6547
C68	O70	C71	117.0861

Table S7: Main bond distances and angles of β-PEF structure

atom1	atom2	distance
C(3)	O(2)	1.2805
C(4)	C(5)	1.3996
C(5)	C(6)	1.401
C(6)	C(7)	1.4073
C(8)	O(4)	1.2831
C(2)	O(1)	1.4592
C(2)	C(9)	1.4438
O(1)	C(3)	1.317
C(3)	C(4)	1.4209
C(3)	O(2)	1.2834
C(4)	C(5)	1.4077
C(4)	O(3)	1.36
C(5)	C(6)	1.4004
C(7)	C(8)	1.4067
C(8)	O(5)	1.3324
C(8)	O(4)	1.2805
O(5)	C(9)	1.3927

atom1	atom2	atom3	angle
C(4)	C(5)	C(6)	105.2689
C(5)	C(6)	C(7)	106.4226
O(1)	C(2)	C(9)	149.9919
C(2)	O(1)	C(3)	108.5823
C(2)	C(9)	O(5)	143.6789
O(1)	C(3)	C(4)	108.5835
O(1)	C(3)	O(2)	125.6216
C(4)	C(3)	O(2)	125.7949
C(3)	C(4)	C(5)	135.1062
C(3)	C(4)	O(3)	113.3985
C(5)	C(4)	O(3)	111.4953
C(4)	C(5)	C(6)	106.4862
C(7)	C(8)	O(5)	108.857
C(7)	C(8)	O(4)	124.8594
O(5)	C(8)	O(4)	126.2836
C(8)	O(5)	C(9)	108.8805
